# Negative cognitive beliefs, positive metacognitive beliefs, and rumination as mediators of metacognitive training for depression in older adults (MCT-Silver)

**DOI:** 10.3389/fpsyg.2023.1153377

**Published:** 2023-03-22

**Authors:** Brooke C. Schneider, Ruth Veckenstedt, Evangelos Karamatskos, Lara Guedes Pinho, Bruno Morgado, César Fonseca, Steffen Moritz, Lena Jelinek

**Affiliations:** ^1^Department of Psychiatry and Psychotherapy, University Medical Center Hamburg-Eppendorf, Hamburg, Germany; ^2^Nursing Department, University of Évora, Évora, Portugal; ^3^Comprehensive Health Research Centre (CHRC), University of Évora, Évora, Portugal; ^4^University Rovira i Virgili, Tarragona, Spain

**Keywords:** metacognitive training, rumination, negative cognitions, older adults, intervention, depression, metacognitive beliefs

## Abstract

**Background:**

Metacognitive Training for Depression in older adults (MCT-Silver; www.uke.de/mct-silver) is a cognitive-behavioral based group intervention that aims at reducing depression by targeting (meta)cognitive beliefs and rumination. In the present study, it was examined whether negative cognitive beliefs, positive metacognitive beliefs and/or rumination may be implicated as mediators of MCT-Silver’s effects on depression.

**Materials and methods:**

We conducted a secondary analysis of a randomized controlled trial comparing MCT-Silver to an active control intervention (cognitive remediation) including 66 older adults (60 years and older) with complete baseline data. Clinician-rated (Hamilton Depression Rating Scale) and self-reported (Beck Depression Inventory-II) depression, negative cognitive beliefs (Dysfunctional Attitudes Scale-18B), positive metacognitive beliefs (positive beliefs subscale; Metacognition Questionnaire-30) and rumination (10-item Ruminative Response Scale) were assessed before (pre) and after 8 weeks of treatment (post), as well as 3 months later (follow-up). It was examined whether change in depression (pre- to follow-up) was mediated by change in negative cognitive beliefs, positive metacognitive beliefs and/or rumination (pre- to post-assessment).

**Results:**

Mediation results differed for self-reported vs. clinician-rated depression. The effect of MCT-Silver on reduction in clinician-rated depression was mediated by a reduction in self-reported rumination, whereas reduction in self-reported depression was mediated by a reduction in negative cognitive beliefs. Positive metacognitive beliefs were not a significant mediator for either outcome.

**Conclusion:**

The current study provides initial evidence for the roles of negative cognitive beliefs and rumination in the treatment of depression in later life with MCT-Silver. Given the divergence of findings and lack of causal precedence, mechanisms of change for MCT-Silver cannot yet be equivocally identified.

## 1. Introduction

Depression is a common mental health disorder in later life such that that up 21.1% older adults (60 years and older) in Europe have clinically relevant depressive symptoms (Hu et al., 2022). Depression is a major cause of disability (Santomauro et al., 2021) and represents a risk factor for dementia (Wu et al., 2020). Underscoring the importance of prompt treatment, depression in older adults has a longer time to remission and is more likely to become chronic compared to depression in younger adults (Schaakxs et al., 2018). Although psychotherapy for depression in older adults is effective (*g* = 0.66; Cuijpers et al., 2014, 2020) and cognitive-behavioral (CBT), interpersonal, reminiscence and problem-solving therapies are recommended by treatment guidelines for depression in older adults (Hinrichsen et al., 2014; DGPPN, 2017), the mediators and mechanisms of change through which these therapies “work” remain unclear (Kazdin, 2007; Cuijpers, 2019). Improved understanding of mediators and mechanisms of change is essential to understanding why and how treatments for depression may work and to improving efficacy of these interventions (Ehring et al., 2022).

Metacognitive Training-Silver (MCT-Silver; www.uke.de/mct-silver) is a low-threshold, CBT-based group intervention developed for older adults with depression. It is based on Metacognitive Training for psychosis (MCT; Moritz et al., 2014), which inspired the development of further MCTs for depression among young and middle-aged adults (D-MCT; Jelinek et al., 2015, 2016, 2019), obsessive–compulsive disorder (Miegel et al., 2021), borderline personality disorder (Schilling et al., 2018), pathological gambling (Gehlenborg et al., 2021), and bipolar disorder (Haffner et al., 2018). Several other metacognition-based interventions have been developed over the past years in addition to MCT (for a review see Moritz et al., 2018). Metacognitive Training adopts the metacognitive perspective (“thinking about thinking”) with the aim of increasing participants’ awareness for cognitive biases, which are the result of depressive information processing styles (e.g., mood-congruent memory such as an older adult focusing exclusively on situations in which they completed a task slower or with more effort). Associated negative cognitive beliefs also represent a target of MCT (“I should not cook anymore if I cannot do it all by myself”). In addition, Metacognitive Training for depression (i.e., D-MCT and MCT-Silver) also addresses problematic coping skills (e.g., rumination about declining ability levels) and metacognitive beliefs about thinking styles (e.g., rumination helps to solve problems). Metacognitive Training utilizes a structured multimedia presentation to convey the content of the intervention and also addresses disorder-specific thought content. Thus, through psychoeducation and interactive exercises, D-MCT and MCT-Silver to improve patients’ awareness of cognitive biases in everyday life. Patients are invited to critically reflect upon thought content and coping skills, which contribute to and maintain depression, and it is discussed how such thoughts and behaviors can be changed. Based on the content of the training, we hypothesize that MCT-Silver could exert its effects on depression through negative cognitive beliefs, positive metacognitive beliefs and/or rumination.

### 1.1. Negative cognitive beliefs

Cognitive theories of depression postulate that a cognitive vulnerability to depression leads to activation of negative cognitive schemas in times of stress and encourages automatic cognitive biases (Beck et al., 1979; Beck and Haigh, 2014). Such biases include, for example, increased attention to and a memory preference for negative information (e.g., mood congruent memory bias) and negative interpretations of ambiguous situations (Mathews and MacLeod, 2005; Moritz et al., 2008). These biases then lead to subsequent endorsement of negative cognitive beliefs about oneself, the world and the future (Nieto et al., 2020). Although cognitive beliefs have been shown to be relatively stable, they are also malleable (Faissner et al., 2018) and the results of several RCTs on CBT interventions have provided evidence of their effects on negative cognitive beliefs (Cristea et al., 2015; Lorenzo-Luaces et al., 2015; Jelinek et al., 2017; Normann and Morina, 2018). However, the role of negative cognitive beliefs in depression treatment has not been equivocally proven. For example, the extent to which changes in such beliefs may occur because of a specific (e.g., CBT-based intervention) versus general (e.g., supportive therapies) treatment or through natural remission of depressive symptoms is unclear (Cristea et al., 2015). Moreover, relatively few studies have examined change in negative cognitive beliefs due to a psychological intervention among older adults.

### 1.2. Positive metacognitive beliefs

MCT-Silver sets a focus on examining one’s thinking processes on the “meta” level. Indeed, it has been suggested that all CBT-based therapies address metacognitive processes as they involve challenging negative thoughts and not accepting thoughts as facts and thus encourage “thinking about one’s thinking” (Moritz et al., 2018). Additionally, Wells has proposed a series of maladaptive metacognitive beliefs about thinking that occur transdiagnostically (Wells and Cartwright-Hatton, 2004). Like negative cognitive beliefs, metacognitive beliefs have also been implicated in the formation and reoccurrence of depression (Papageorgiou and Wells, 2009; Weber and Exner, 2013; Faissner et al., 2018). Differing from negative cognitive beliefs, metacognitive beliefs are focused, for example, on the usefulness, dangerousness and/or controllability of thinking rather than thought content. The Metacognition Questionnaire-30 (Wells and Cartwright-Hatton, 2004) is often used to assess metacognitive beliefs on five subscales: cognitive confidence, positive beliefs about worry, cognitive self-consciousness, negative beliefs about the uncontrollability of thoughts and danger, and beliefs about the need to control thoughts. Specifically, positive metacognitive beliefs refer to beliefs regarding the usefulness of rumination in solving problems and overall coping. We hypothesize that among Wells’ metacognitive domains, positive cognitive beliefs would be implicated in the effects of MCT-Silver as the training content directly addresses assumptions regarding the usefulness of rumination. Moreover, paying more attention to thoughts (e.g., to reframe them) and reducing confidence in (negative) beliefs and biases (e.g., mood-congruent memory) or catastrophic assumptions (e.g., jumping to conclusions) is rather encouraged in MCT-Silver and, therefore, is not in line with Wells’ concept.

### 1.3. Rumination

Rumination has been conceptualized in varying ways. According to the Response Styles Theory (Nolen-Hoeksema, 1987), rumination represents a transdiagnostic emotion regulation (ER) strategy involving repetitively and passively focusing on negative feelings, symptoms of distress and their meaning and consequences (Aldao et al., 2010). ER has been defined as a conscious or unconscious and automatic or controlled attempt to increase or decrease an emotional experience (for a review see Braunstein et al., 2017) in order to appropriately respond to environmental demands (Campbell-Sillis and Barlow, 2007; Berking and Wupperman, 2012). Thus, rumination may be described as an automatic ER response conditioned to triggering stimuli, such as low mood (Watkins and Nolen-Hoeksema, 2014), which involves self-reflection and self-focused attention (Lyubomirsky and Nolen-Hoeksema, 1993). Goals of rumination may include attempts at problem-solving or gaining clarity regarding symptoms (e.g., “Why do I have these symptoms?”). Although metacognitive beliefs are associated with rumination, several additional causal factors must be considered when understanding which patients use it as an emotion regulation strategy. For example, learning processes based on experiences with parents who demonstrate passive coping styles, as well as an overly critical and controlling parental style have been associated with increased use of rumination. Also, cognitive (e.g., evaluation of discrepancies between actual and desired states), genetic and neurocognitive factors (e.g., reduced cognitive control) are implicated in rumination (for a review see Watkins and Roberts, 2020).

Rumination is linked to a plethora of negative psychological consequences, including increasing the severity of and prolonging negative mood and associated negative thinking, and impairing problem-solving and engagement in positive behaviors (Watkins and Roberts, 2020). Supporting its role as a (primarily maladaptive) ER strategy separate from depressive symptoms, rumination predicts the onset of major depressive episodes (Nolen-Hoeksema, 2000; Watkins and Roberts, 2020) and rumination following a stressor is associated with greater depression severity, in both cases also after accounting for baseline levels of depressive symptoms (Michl et al., 2013). Moreover, in a study utilizing ecological momentary assessment, rumination mediated longitudinal relationships between stress and both negative affect and depressive symptoms for stressful events occurring in daily life (Ruscio et al., 2016).

Whereas repetitive thinking in and of itself may not worsen mood (Emery et al., 2020), particularly a repetitive focus on negative thoughts, such as those involved in Beck’s cognitive triad, are implicated in reduced mood due to rumination (Ehring and Watkins, 2008; Poerio et al., 2014). CBT has moderate effects on rumination (Hedge’s *g* = 0.57; Spinhoven et al., 2018); however, rumination has been less frequently included as an outcome in RCTs. Although rumination has also been implicated in depression among older adults (Tang et al., 2022), older adults ruminate less frequently than their younger counterparts (Nolen-Hoeksema and Aldao, 2011; Ricarte et al., 2016) and age-related differences in ER have been well documented (Carstensen et al., 1999; Emery et al., 2020). Thus, findings from studies with younger or middle-aged adults should not be generalized to older adults. Evidence for the effects of psychological interventions on rumination in older adults remains insufficient (Spinhoven et al., 2018). We found only one study examining the effects of a CBT-based intervention on rumination among older adults (Ekkers et al., 2011).

### 1.4. Empirical findings on metacognitive training for depression

MCT-Silver represents an age-adapted version of D-MCT and while there is significant overlap between the content of the two treatments (Jelinek et al., 2015), MCT-Silver includes unique content, which is described below. An initial RCT on D-MCT yielded medium to large effects on clinician- and self-reported depressive symptoms, as well as small to medium effects on negative cognitive beliefs following the intervention as well as after 6-months among young and middle-aged adults participating in an outpatient rehabilitation program (Jelinek et al., 2016). Moreover, D-MCT had moderate to large effects on metacognitive beliefs immediately following the intervention (Jelinek et al., 2017). There is also evidence of maintenance of these changes after 3 years (Jelinek et al., 2019). In separate studies, a superior effect of D-MCT as an add-on intervention among patients completing an intensive inpatient program was found for negative cognitive beliefs (Hauschildt et al., 2022) and in a study with outpatients, significantly greater reductions in rumination and metacognitive beliefs were found in the D-MCT group compared to a wait-list control (Özgüç and Tanriverdi, 2022). In an initial examination of the relative contributions of metacognitive vs. cognitive processes to depression reduction in D-MCT, Jelinek et al. (2017) compared negative cognitive beliefs with three subscales of the Metacognitive Questionnaire (MCQ; ‘need for control’, ‘negative beliefs’, ‘positive beliefs’). Only improvement on the ‘need for control’ subscale of the MCQ significantly mediated D-MCT’s effect on reduction in depressive symptoms at a medium effect.

The acceptance and feasibility of metacognitive training with older adults was confirmed in a pilot study in which D-MCT groups were offered as an add-on treatment to older adults participating in an intensive inpatient treatment program (Schneider et al., 2018). In a revision phase, based on patient feedback and empirical findings on depression in later life, select modules and exercises were revised. Specifically, a D-MCT module on self-esteem was altered to address negative attitudes about aging (Chachamovich et al., 2008; Laidlaw et al., 2018) and an imagery rescripting exercise regarding self-image replaced several D-MCT exercises. A second module was partially revised to integrate concepts from acceptance and commitment therapy (ACT) in the context of accepting negative feelings with a focus on age-related changes (e.g., mobility limitations, illness, loss of significant others). Finally, a new module was developed to address (re-)defining values later in life (e.g., due to changing roles and priorities). All case examples were revised as necessary for older adults and the format was edited to improve the clarity and presentation of the content based on patient feedback. Therefore, MCT-Silver is best conceptualized as a “modern” CBT-based intervention, which integrates traditional CBT with third-wave techniques and theories utilizing an overarching metacognitive approach.

In a recently completed RCT comparing MCT-Silver to an active control group (cognitive remediation; Schneider et al., *under review*), both groups had large and significant reductions on the primary outcome (Hamilton Depression Rating Scale; HDRS) from baseline to post (*t*1) and follow-up (*t*2; *d_MCT-Silver_* = 1.26–1.42; *d*_CR_ = 1.05–1.12). However, there were no significant group differences (*η*_p_^2^ = 0.001–0.002). For self-reported depression (Beck Depression Inventory – II; BDI-II) and rumination (Ruminative Response Scale; RRS), MCT-Silver yielded significant moderate to large effects compared to CR immediately following the intervention (*t*1) and after 3 months (*t*2; BDI-II: *η*_p_^2^ = 0.075–0.135; RRS: *η*_p_^2^ = 0.087–0.127). A significant moderate effect was found for positive metacognitive beliefs (MCQ-PB) at post-assessment (*t*1; *η*_p_^2^ = 0.067), but group differences did not reach significance at follow-up (*t*2: *η*_p_^2^ = 0.027). The MCT-Silver group had small to moderate reductions in negative cognitive beliefs (Dysfunctional Attitudes Scale-18B) at post- and at 3-month follow-up (*d_MCT-Silver_* = 0.24–0.30), whereas negative cognitive beliefs remained stable in the CR group (*d_CR_* = −0.06–0.06).

### 1.5. Aim of the present study

The mediators through which MCT-Silver exerts its effects have not yet been examined. By including negative cognitive beliefs, positive metacognitive beliefs and rumination as mediators, we sought to explore whether MCT-Silver’s impacts on depression relative to an active control condition (i.e., cognitive remediation; MyBrainTraining©; NeuroCare GmbH) may be due to specific (meta)cognitive mechanisms (e.g., changes in negative cognitive beliefs or positive metacognitive beliefs), to improvement in an ER strategy (e.g., rumination) or both. To this end, we conducted a secondary analysis of data from our recent RCT on the effectiveness of MCT-Silver. In line with the previously presented theoretical models and work on the effects of CBT and metacognitive-based interventions, we expected that a better outcome at 3-month follow-up after MCT-Silver treatment would be mediated by an improvement in negative cognitive beliefs (DAS-18B), positive metacognitive beliefs (MCT-PB) and rumination (RRS) from baseline to post-intervention. Although the RCT did not yield significant between-group differences for clinician-rated depression, based on current recommendations regarding probing for mediation effects in the absence of group differences (MacKinnon et al., 2007; Hayes, 2022, p. 123), we examined mediators of reduction in both clinician-rated and self-reported depressive symptoms. Due to links between late-onset depression and neurodegenerative changes (Leyhe et al., 2017), we sought to control for possible confounds by additionally examining models including covariates (late / early onset of depression as indicated by self-reported depressive symptoms prior to age 60 and number of depressive episodes).

## 2. Materials and methods

### 2.1. Design

The present study represents a secondary analysis of data from an RCT comparing group MCT-Silver for older adults with depression to an active control (e.g., cognitive remediation). All participants were assessed at three time points: baseline (*t*0), post (*t*1; 8 weeks) and follow-up (*t*2; 3 months after post). After t0, participants were randomized to one of the two groups. Study leads performed the randomization using a randomization plan developed by a statistician (1,1 allocation rule). Raters were blinded to group allocation. To ensure rater blindness throughout the study, raters reminded participants at the beginning of the post- and follow-up assessments not to disclose their group assignment. Informed consent was obtained before the interview from all participants. Participants were given 20€ upon completion of each assessment as compensation for their time and travel costs. The study received ethical approval and was registered at Clinical Trials.gov (NCT03691402). The trial was conducted in accordance with the Declaration of Helsinki.

### 2.2. Participants and procedure

We recruited participants *via* Google AdWords, articles in a senior magazine, posters, brochures, advertisements, as well as through depression, anxiety and memory outpatient clinics. Prior to the baseline examination, potential participants were screened for eligibility in a telephone interview and then an appointment was made for the baseline examination. Before participants attended interviews, they completed measures of secondary outcomes sent *via* post. The Mini International Neuropsychiatric Interview (MINI; German version, 7.0.2; Sheehan et al., 1998) was used to assess for a current major depressive episode, recurrent depression and/or dysthymia (inclusion criteria). Additionally, participants had to (1) be at least 60 years old, (2) provide consent to participate in MCT-Silver as well as in the diagnostic interviews, (3) be available for weekly group sessions, (4) be eligible for group therapy (ability to generally comply with group rules was assessed during the screening interview), (5) have sufficient German language skills, and (6) score within the intact range (≥17 points) on a telephone version of the Mini Mental State Examination (ALFI-MMSE; Roccaforte et al., 1992). Exclusion criteria were as follows: (1) current or lifetime psychotic symptoms, (2) current or lifetime mania, (3) severe neurological disease (e.g., Parkinson’s disease, multiple sclerosis), (4) current substance dependence, (5) visual or hearing impairment, which prevented group participation and/or testing, and/or (6) current acute suicidality. Current substance use or abuse was tolerated. Concurrent outpatient psychotherapy or pharmacological treatment did not lead to exclusion from the study but was carefully documented.

### 2.3. Implementation of interventions

#### 2.3.1. Experimental intervention: Metacognitive training–Silver

One MCT-Silver session was administered per week over a period of 8 weeks (*ca.* 60 min per session). MCT-Silver groups were conducted by licensed psychotherapists as well as psychologists with master’s degrees who were currently undergoing postgraduate training in psychotherapy. Two trainers led each group. All trainers received training in MCT-Silver prior to their participation in the study and were regularly supervised by one of the study leads (BS, RV for an MCT-Silver and D-MCT Online Training see www.uke.de/e-dmct). All MCT-Silver modules were presented as slides that contain the training content. The number of participants in MCT-Silver groups ranged from three to eight; due to the open format of MCT-Silver, participants could join at any time. The modules cover topics including negative cognitive beliefs, metacognitive beliefs, rumination and depressive behaviors, which are supported by significant research on depression: Modul 1: Mental filter (Carver and Ganellen, 1983; Gotlib and Joormann, 2010); Module 2: Mood-congruent memory / false memories (Mathews and MacLeod, 2005; Moritz et al., 2008), Module 3: “Should” statements (Graham et al., 2010; Egan et al., 2011) and disqualifying the positive (Cane and Gotlib, 1985; Elliott et al., 1997) as well as acceptance of negative feelings (Hayes et al., 1996; Butler and Ciarrochi, 2007); Module 4: Values (Isaacowitz and Seligman, 2002; Hayes et al., 2006; Wrosch et al., 2013); Module 5: Exaggeration/Minimization (Garber and Hollon, 1980; Hoehn-Hyde et al., 1982; Cane and Gotlib, 1985; Wenzlaff and Grozier, 1988) as well as Attribution Style (Carver and Ganellen, 1983; Wenzlaff and Grozier, 1988), Module 6: Rumination and Withdrawal (Rood et al., 2009; Seidel et al., 2010; Wells, 2011); Module 7: Jumping to Conclusions (Strunk et al., 2006; Miranda et al., 2008), and Module 8: Self-Worth in Later Life (Davey et al., 2004; Orth et al., 2009; Holmes et al., 2016).

#### 2.3.2. Control intervention: Cognitive remediation

An active control condition was administered to match the treatment group in terms of therapeutic effort. Participants in the control group completed MyBrainTraining© exercises on a computer once a week for up to 60 min. Although participants often completed the training in groups of 2–3, some sessions were individually scheduled. Training sessions were conducted in the hospital where the study took place. Psychologists were present to mark attendance, monitor for worsening of symptoms and help with possible computer problems, but did not administer any structured intervention. Log-in information was not given to participants to prevent practice at home.

### 2.4. Measures

Two outcomes were investigated. The primary outcome measure was the total score of the Hamilton Depression Rating Scale (HDRS, 17-item version; Hamilton, 1960). The secondary outcome measure was self-rated depression as measured by the Beck Depression Inventory-II (BDI-II; Beck et al., 1996).

#### 2.4.1. Potential cognitive mediators

##### 2.4.1.1. Dysfunctional Attitudes Scale-18B (DAS-18B)

The Dysfunctional Attitudes Scale is a self-report questionnaire designed to assess and identify dysfunctional attitudes, thoughts, and schemas associated with depression. For the present study, the German DAS-18B (Rojas et al., 2015) was used, which consists of 18 items answered on a seven-point Likert scale (1 = total agreement to 7 = total disagreement). A higher total score indicates a greater presence of dysfunctional attitudes. Internal consistency for our study was good (Cronbach’s *α* = 0.85).

##### 2.4.1.2. Positive beliefs subscale, Metacognitions Questionnaire-30 (MCQ-30)

The Metacognitions Questionnaire-30 is a self-rating questionnaire developed by Wells and Cartwright-Hatton (2004). The questionnaire has good psychometric properties with a retest reliability of *r* = 0.75 and internal consistency of *α* = 0.72. Internal consistency of the positive beliefs subscale in the current sample was acceptable (Cronbach’s *α* = 0.79).

##### 2.4.1.3. 10-item Ruminative Response Scale

The 10-item Ruminative Response Scale (Treynor et al., 2003) is a subscale of the Response Style Questionnaire (Nolen-Hoeksema and Morrow, 1991), which assesses ruminative tendencies. The scale contains only items unconfounded with depression. The RRS-10 has demonstrated high internal consistency and test–retest reliability (Treynor et al., 2003). Internal consistency in our study was also good (Cronbach’s α = 0.82).

### 2.5. Strategy of data analysis

IBM SPSS 27.0 software was used for all analyzes. A subsample of participants with complete baseline data were included for this secondary analysis (*N* = 66). Participants with missing baseline data did not differ from those with complete baseline data regarding demographic or clinical characteristics (age, gender, education, number of depressive episodes, baseline scores on the HDRS or BDI-II). There were also no significant differences for change on depression measures over the study period (*t*0-*t*2) or mediators (*t*0-*t*1). Missing outcome data (post, follow-up) were imputed by the expectation–maximization (EM) algorithm trimmed to fall between the minimum and maximum of possible values. Although various cutoffs have been defined for determining early- and late-onset of depression in the literature, we defined early-onset depression as self-reported depressive symptoms prior to age 60. For mediation analysis, MCT-Silver was coded as 1 and CR as 0. A treatment effect in the analyses thus refers to effects of MCT-Silver above and beyond CR. To capture change in depression (HDRS and BDI-II), standardized residualized change scores using a simple linear regression model in which baseline scores predicted follow-up scores (*t*0 to *t*2) were calculated. To capture change in the mediators (DAS-18B, MCQ-PB and RRS), standardized residualized change scores were calculated in which baseline scores predicted post-assessment scores (*t*0 to *t*1). Greater declines in the respective variable is indicated by more positive standardized residualized change scores. The mediation analysis thus determines to what extent change in depression from *t*0 to *t*2, i.e., from baseline to 3-month follow-up, that was brought about by MCT-Silver above and beyond CR can be explained by change in the mediator variables in the treatment period, i.e., from baseline to end of treatment 8 weeks later (*t*0-*t*1). We expected that the mediation analysis would yield positive beta values for path a (group to mediator), which would indicate that MCT-Silver led to a greater reduction in the mediator versus CR. Path b would also result in a positive beta value (mediator to HDRS) indicating that a reduction in the mediator led to a reduction in depression. The mediation analysis was conducted using an SPSS macro PROCESS developed by Hayes (2022). The analysis allows delineation of the effects of each of the proposed mediators separately while controlling for the others. It thus presents a rather conservative test for estimating individual mediation effects. To correct for potential biases of non-normality in the sample, results were bootstrapped 5,000 times. When the effect range (LL = lower limit to UL = upper limit) of the 95% CI does not include zero, the null hypothesis is considered rejected.

## 3. Results

Groups were similar on psychopathological and sociodemographic data as well as medication (see Table 1). Most participants (*n* = 56; 84.9%) met criteria for a current depressive episode. Six (9.1%) participants fulfilled criteria for recurrent depression without a current major depressive episode or dysthymia (all also met the HDRS cutoff for at least mild depression according to the HDRS [≥9]; DGPPN, 2017). Approximately one-third of participants (34.9%; *n* = 23) had a single depressive episode whereas 60.6% (*n* = 40) had recurrent depressive episodes. Half (*n* = 33; 50.0%) fulfilled criteria for dysthymia. Spearman correlations between change in all variables over the different time points (*t*0-*t*1; *t*0-*t*2) are displayed in Table 2. As expected, correlations were highest within the same constructs over time (change on BDI-II from t0-t1 and t0-t2) but change in DAS-18B, RRS and MCQ-PB were also significantly correlated. Change in MCQ-PB was not significantly correlated with change in depression at any time point.

**Table 1:** Baseline sociodemographic and clinical characteristics: Means (SD) or frequencies.

Variable	MCT-Silver (*n* = 34)	CR (*n* = 32)	Statistics (MCT-Silver vs. CR)
Age (years)	72.29 (6.41)	71.53 (7.40)	*t*(64) = 0.60, *p* = 0.655
Sex (female/male)	26/8	22/10	*χ*^2^ = 0.50, *p* = 0.482
Formal education (years)	10.21 (1.82)	10.53 (1.76)	*t*(64) = 0.74, *p* = 0.464
Marital status (single/married/separated or divorced/widowed)	7/6/12/9	5/8/11/8	Cramer’s *V* = 0.10, *p* = 0.882
Number of MDE (incl. current)	3.85 (4.45)^a^	3.93 (3.28)^b^	*t*(61) = 0.44, *p* = 0.932
Onset of depression (early vs. late)*	19/15	16/16	*χ*^2^ = 0.29, *p* = 0.632
Medication (antidepressant/benzodiazepine/combination/none/other)	8/2/3/20/1	14/1/0/16/1	Cramer’s V = 0.29, *p* = 0.252
Comorbidity (MMD only/MDD and comorbid disorder)	24/10	21/11	*χ*^2^ = 0.19, *p* = 0.665
Psychopathology			
HDRS-17	17.30 (4.75)	15.44 (4.06)	*t*(64) = 0.81, *p* = 0.096
BDI-II	29.88 (9.38)	27.57 (10.04)	*t*(64) = 0.97, *p* = 0.336
DAS-18B	62.06 (21.02)	54.84 (17.40)	*t*(64) = 0.29, *p* = 0.135
RRS	24.75 (5.65)	23.25 (5.25)	*t*(64) = 0.60, *p* = 0.267
MCQ-PB	12.55 (4.27)	11.44 (3.37)	*t*(64) = 1.17, *p* = 0.245

*Early-onset depression was defined as self-reported depressive symptoms prior to age 60. BDI-II = Beck Depression Inventory-II; CR = Cognitive remediation; DAS-18B = Dysfunctional Attitudes Scale, Version 18-B; HDRS = Hamilton Depression Rating Scale; MCQ-PB = Metacognition Questionnaire-Positive Beliefs subscale; MDE = Major depressive episodes; RRS = Ruminative Response Scale; ^a^ *n* = 33, ^b^ *n* = 30.

**Table 2:** Pearson’s correlations between change in self-reported and clinician-rated depression with change in negative cognitive beliefs, positive metacognitive beliefs and rumination (*N* = 66).

	1.	2.	3.	4.	5.	6.	7
1. ΔBDI-II, t0-t1	--	0.415***	0.508***	0.488***	0.103	0.698***	0.313*
2. ΔHDRS, t0-t1		--	0.247*	0.402***	0.047	0.403**	0.642**
3. ΔDAS-18B, t0-t1			--	0.434***	0.368**	0.433***	0.057
4. ΔRRS, t0-t1				---	0.281*	0.323**	0.297*
5. ΔMCQ-PB, t0-t1					---	0.084	−0.023
6. ΔBDI-II, t0-t2						---	0.403***
7. ΔHDRS, t0-t2							---

BDI-II = Beck Depression Inventory-II; DAS-18B = Dysfunctional Attitudes Scale, Version 18-B; HDRS = Hamilton Depression Rating Scale; RRS = Ruminative Response Scale; MCQ-PB = Metacognition Questionnaire-Positive Beliefs subscale. *p < 0.05; **p < 0.01; ***p < 0.001.

Mediation was tested separately for the HDRS and BDI-II (Table 3). Consistent with the main RCT results, MCT-Silver elicited long-term reduction in depression only on the BDI-II (*b* = 0.70, SE = 0.22, *p* = 0.002, BootLLCI = 0.26, BootULCI = 1.14), whereas the treatment group effect was not significant for the HDRS (*b* = −0.03, SE = 0.24, *p* = 0.902, BootLLCI = −0.50, BootULCI = 0.45). Treatment with MCT-Silver elicited a greater reduction on the MCT-PB and RRS as well as the DAS-18B at post-intervention. Mediation results differ for the two depression measures: For the HDRS, the indirect effect of treatment on reduction in clinician-rated depression was significant *via* RRS (*b* = 0.18; BootSE = 0.12; BootLLCI = 0.01, BootULCI = 0.46) with a non-significant effect of treatment on reduction in depression of −0.14 (SE = 0.25, *p* = 0.570, BootLLCI = −0.63, BootULCI = 0.35). For the BDI-II, the indirect effect of treatment on reduction in self-reported depression was significant *via* DAS-18B (*b* = 0.19, BootSE = 0.10, BootLLCI = 0.02, BootULCI = 0.41) with a remaining significant effect of treatment on reduction in depression of 0.51 (SE = 0.22, *p* = 0.022, BootLLCI = 0.08, BootULCI = 0.95). For the non-significant indirect effects of the MCQ-PB for both depression measures as well as the DAS-18B and RRS for the HDRS and BDI-II, please see Table 4.

**Table 3:** Direct effects of treatments on mediators and of mediators on clinician-rated and self-reported depression.

	Mediators (*t*0-*t*1)					Outcomes (*t*0-*t*2)
	DAS-18B		RRS		MCQ-PB		HDRS		BDI-II	
	b	SE	b	SE	b	SE	b	SE	b	SE
Treatment group	0.57*	**0.24**	0.51*	**0.24**	0.50*	**0.23**	−0.14	0.25	0.51*	**0.22**
DAS-18B							−0.04	0.13	0.33**	**0.12**
RRS							0.35*	**0.13**	0.14	0.12
MCQ-PB							−0.09	0.13	−0.15	0.12
*R* ^2^	0.08		0.07		0.07		0.11		0.29	

BDI-II = Beck Depression Inventory-II; DAS-18B = Dysfunctional Attitudes Scale, Version 18-B; HDRS = Hamilton Depression Rating Scale; RRS = Ruminative Response Scale; MCQ-PB = Metacognition Questionnaire-Positive Beliefs subscale. **p < 0.01; *p < 0.05. Treatment group coded as MCT-Silver = 1 and active control = 0. Higher scores on the mediators and outcomes indicate greater reductions. Bold indicates that the 95% confidence interval does not encompass zero.

**Table 4:** Indirect effects of potential mediators on self-reported and clinician-rated depression.

	HDRS (*t*0-*t*2)				BDI-II (*t*0-*t*2)			
	Effect	BootSE	BootLLCI	BootULCI	Effect	BootSE	BootLLCI	BootULCI
DAS-18B	−0.02	0.08	−0.20	0.13	**0.19**	**0.10**	**0.28**	**0.40**
RRS	**0.18**	**0.12**	**0.01**	**0.45**	0.07	0.07	−0.04	0.25
MCQ-PB	−0.04	0.08	−0.23	0.10	−0.08	0.08	−0.25	0.06
Total	0.11	0.13	−0.14	0.38	0.18	0.12	0.44	−0.02

BDI-II = Beck Depression Inventory-II; DAS-18B = Dysfunctional Attitudes Scale, Version 18-B; HDRS = Hamilton Depression Rating Scale; RRS = Ruminative Response Scale; MCQ-PB = Metacognition Questionnaire-Positive Beliefs subscale. Bold indicates that the 95% confidence interval does not encompass zero.

With regard to the covariates early−/late-onset and number of depressive episodes, participants reporting early-onset of depressive symptoms (prior to age 60) had, on average, 5.25 (SD = 4.59) depressive episodes, whereas those with late-onset had 2.48 (SD = 2.39) depressive episodes (*t*(46.99) = 3.01; *p* < 0.004). Mediation was again tested separately for the HDRS and BDI-II when including the covariates age of onset and number of depressive episodes. Indirect effects of mediators were not substantially changed. For the HDRS, the indirect effect of treatment on reduction in clinician-rated depression remained significant only *via* RRS (*b* = 0.21; BootSE = 0.13; BootLLCI = 0.01, BootULCI = 0.53) with a non-significant effect of treatment on reduction in depression of −0.14 (SE = 0.26, *p* = 0.60, BootLLCI = −0.66, BootULCI = 0.39). For the BDI-II, the indirect effect of treatment on reduction in self-reported depression remained significant only *via* DAS-18B (*b* = 0.16, BootSE = 0.09, BootLLCI = 0.01, BootULCI = 0.36) with a significant effect of treatment on reduction in depression of 0.48 (SE = 0.22, *p* = 0.033, BootLLCI = 0.04, BootULCI = 0.93).

## 4. Discussion

Our study provides first evidence of how MCT-Silver may exert its effects on depression. In this secondary analysis of our RCT data, MCT-Silver led to a significant reduction in rumination, positive metacognitive beliefs and negative cognitive beliefs above and beyond CR. However, our results regarding mediation are not fully conclusive. Whereas rumination mediated reduction in clinician-rated symptoms, negative cognitive beliefs mediated the effect of MCT-Silver on reduction in self-reported depression. Conclusions regarding causal mechanisms for MCT-Silver are also limited by the lack of causal precedence given that there was an overlap in the measurement of mediators and outcomes. Thus, although our findings support significant statistical mediation, causation remains unclear (Kazdin, 2007). We first discuss the overall findings with regard to their relevance as possible mechanisms of change in MCT-Silver and then address possible reasons for the discrepancy between the BDI-II and HDRS.

Consistent with the results from our recent RCT on MCT-Silver, we did not find a direct effect of MCT-Silver on the main outcome–reduction in clinician-rated depressive symptoms (HDRS). However, the indirect effect of MCT-Silver *via* RRS on depressive symptoms was significant. Failure to test for indirect effects despite the absence of significant total or direct effects in mediation models may lead to an under analysis of data and missed identification of meaningful mediation effects (Hayes, 2022, p. 123). Given the significant impairment associated with rumination, this ER strategy represents an important target for psychological interventions. Our findings indicate that the reduction in rumination from pre- to post-assessment due to MCT-Silver significantly mediated the reduction in depressive symptoms for clinician-assessed depression from pre-assessment to the 3-month follow-up. In other words, MCT-Silver exerted its effect on reduction in clinician-rated depression symptoms only *via* reduced ruminations.

Given that the overarching goal of MCT-Silver is gaining insight into thought processes (e.g., “thinking about one’s thinking”), it is likely that several of the cognitive and metacognitive techniques (e.g., questioning the usefulness of rumination, challenging negative thoughts that serve as ruminative content, not treating thoughts as facts) and general psychoeducation (e.g., differences between problem-solving and rumination) presented in MCT-Silver contributed to reduced ruminations (Papageorgiou and Wells, 2009; Hawley et al., 2014). In addition, MCT-Silver integrates other approaches, which may have impacted rumination. For example, with the aim of increasing awareness of cognitive biases, MCT-Silver Module 2 “Memory” specifically addresses a tendency to remember situations from the past overly positively or negatively. Patients are then encouraged to seek more specific, accurate memories and to remain “fair” when comparing their current life with the past. Thus, increased awareness of mood-congruent memory biases may lead to reduced ruminations. Alternatively, similar to reminiscence therapy, training patients to search for more specific autobiographical memories may improve specificity of information processing (Hamlat, 2018). Moreover, integrating concepts from third-wave treatments, in Module 3, increasing acceptance for negative feelings is presented. It is then discussed that acceptance of and giving up fighting negative thoughts and feelings can lead to improved mood. This would then fit well with Nolen-Hoeksema’s et al. (2008) concept of shifting attention away from negative thought content.

Although our findings on rumination are promising, the literature is not equivocal regarding to what extent rumination may be best conceptualized as an ER strategy versus as a component of an emotional reaction to an ongoing emotional conflict (e.g., depressive symptoms and their causes). Similar to other components of emotional reactions (e.g., physiological, cognitive), rumination begins automatically and involuntarily. Thus, its measurement *via* self-report measures has been criticized as it is unclear to what extent individuals can accurately self-report on such strategies as reflecting upon one’s emotion regulation requires significant insight and metacognitive ability (Aldao et al., 2010; Berking and Wupperman, 2012). Moreover, the experience of rumination may be confounded with cognitive-emotional aspects of depression itself (e.g., negative thoughts associated with depression or thinking about feelings of sadness; Aldao et al., 2010). Studies demonstrating qualitative differences in depressed and non-depressed individuals regarding rumination may provide supporting evidence for this view. For example, depressed individuals have more thoughts regarding hopelessness while ruminating and feel more sadness during rumination than non-depressed individuals (Rosenbaum et al., 2020). To increase specificity of measurement of depression versus rumination in our study, we used the 10-item RRS which was developed to reduce overlap between rumination and depressive symptoms (Treynor et al., 2003).

To date, the effects of D-MCT on rumination has only been considered in two studies (Jelinek et al., 2013; Özgüç and Tanriverdi, 2022), which yielded significant reductions in rumination at small (Cohen’s *d* = 0.32) and large (*η*2 = 0.229) effects, respectively. Further work is needed to examine whether these findings can be replicated in future studies and specifically for MCT-Silver in older adults. Taken together, in line with general CBT concepts for depression treatment, our study adds further evidence that rumination represents an important treatment target also for older adults with depression, is malleable and can mediate a reduction in depressive symptoms among this patient group.

In line with cognitive models of depression, reduction in negative cognitive beliefs because of MCT-Silver mediated reduction in self-reported depression. As a CBT-based intervention, MCT-Silver aims to improve participants’ awareness of negative thought patterns and cognitive biases. In a second step, participants are taught over several modules how such negative thoughts can be challenged and modified to be more realistic and “fair.” The impact of these negative thoughts on mood is also highlighted in the training. The current study thus provides further support for the malleability of negative cognitive beliefs among older adults, which to date has been shown to be important for depression recovery primarily within younger and middle-aged adults (Cristea et al., 2015; Faissner et al., 2018).

Our results differ somewhat from previous work on D-MCT, in which the DAS did not emerge as a significant mediator; rather the ‘cognitive control’ subscale of the MCQ-30 was the only significant mediator (Jelinek et al., 2017). Given that we did not include the same MCQ subscales in the current study, these mediation models cannot be directly compared. Although MCT-Silver includes content similar to D-MCT, specific treatment content does differ (e.g., the inclusion of components drawn from ACT in MCT-Silver). Therefore, it is not surprising that the mediators through which the treatments affect depression also differ (at least with regard to the DAS-18B). It is also unclear to what extent age-related differences, for example, in emotion processing or attentional biases, may impact the (purported) mechanisms through which D-MCT and MCT-Silver work.

The discrepant findings between self- and clinician-reported depression, prohibits a conclusive statement regarding mediators of the effects of MCT-Silver on depressive symptoms. Moreover, given the overlap of measurement periods for outcomes and mediators, causality of these associations also cannot be equivocally established. Therefore, our study provides initial evidence that MCT-Silver may work through reduction of specific cognitive processes (e.g., reduction of negative cognitive beliefs) as well as through improving ER strategies (e.g., rumination). Discrepancies between the BDI-II and HDRS are not uncommon. Change on the BDI-II and HDRS in our study were significantly correlated (*r* ≈ 0.40); however, correlations between the BDI-II and HDRS have been shown to vary widely (*r* = 0.20–0.89; Schneibel et al., 2012). Moreover, the HDRS has been criticized for underestimating cognitive symptoms of depression (Zimmerman et al., 2005), which in our study is reflected by the smaller and non-significant associations between change on the DAS-18B and the HDRS at post and follow-up, respectively (see Table 2). Thus, the specific content of the DAS-18B may better correspond to the cognitive aspects of depression measured by the BDI-II.

Finally, our findings were unchanged after controlling for possible effects of late−/early-onset of depression as well as number of depressive episodes. This is broadly in line with studies indicating that effects of depression treatment do not differ based on age of onset (Kozel et al., 2008; Tunvirachaisakul et al., 2018). However, older adults with both late-onset and recurrent depression may take longer to achieve remission (Driscoll et al., 2005). Given that our measures were based on self-report data, future work utilizing neuroimaging data may better rule out effects of neurodegenerative processes.

### 4.1. Limitations

The current study has several strengths (e.g., RCT, blinded assessors, confirmed diagnoses, analyses controlled for possible confounding variables) and sheds further light on mediators of reduction in depressive symptoms within older adults, but it is not without its limitations. First, it is important to emphasize that this study represents an analysis of a subsample of our RCT participants. Thus, our conclusions only pertain to this specific sample and there were some minor differences on secondary outcomes (e.g., significance of between-group reductions in DAS-18B at post-assessment). Next, as previously mentioned, we cannot draw equivocal conclusions given that mediators differed for self-reported versus clinician-rated depression. Third, participants had mild to moderate depressive symptoms, and it is unclear whether our findings may apply to patients with more severe depression. Interestingly, self-rated depression on the BDI-II was more severe compared to clinician ratings of depressive symptoms. Patients reported having on average four depressive episodes and half (*n* = 33) met criteria for dysthymia. This suggests that many participants were experiencing perhaps milder yet chronic symptoms.

Although our study’s mediation models represent methodological standards for mediation (Hayes, 2022), future work should seek to provide further support for MCT’s mechanisms of change (Kazdin, 2007). The ideal test of mediation would include intermediate measurements during treatment as well as a manipulation of these mediators (Ehring et al., 2022). For example, it would be ideal to measure changes in possible mediators following each session or at intermediate points throughout treatment (e.g., Lemmens et al., 2017). Given that our study only included three time points and the period of measurement of mediators and outcomes overlapped, this prohibits the investigation of causal relationships between rumination, negative cognitive beliefs and metacognitive beliefs with each other as well as with regard to change in depressive symptoms. Our study examined reductions in depressive symptoms at follow-up, which also captures maintenance of intervention effects. Finally, it is important to point out that mediation only provides evidence regarding which of the tested variables account for change due to treatment and do not exclude the possibility that other factors (e.g., also including common factors, other ER strategies, knowledge) contributed to the observed reductions in depressive symptoms. Specifically, we did not include measures associated with the integrated ACT techniques.

### 4.2. Conclusion

Taken together this study provides initial evidence for negative cognitive beliefs and rumination as mechanisms of change in MCT-Silver. These findings underscore the importance of targeting negative cognitive beliefs and rumination when treating depression among older adults, however, given the divergent outcomes for self-reported and clinician-rated outcomes as well as the lack of casual precedence, MCT-Silver’s mechanisms of change have yet to be conclusively identified.

## Data availability statement

The raw data supporting the conclusions of this article will be made available upon reasonable request without undue reservation.

## Ethics statement

The studies involving human participants were reviewed and approved by Local Psychology Ethics Commission (Lokale Psychologische Ethikkomission) of the Center for Psychosocial Medicine at the University Medical Center Hamburg-Eppendorf. The patients/participants provided their written informed consent to participate in this study.

## Author contributions

BS: conceptualization, methodology, formal analysis, writing the original draft, and funding acquisition. RV: supervision, investigation, conceptualization, and project administration. EK: writing–review and editing, methodology, and resources. LP, BM, and CF: writing–review and editing, and resources. SM and LJ: conceptualization, methodology, resources, and writing–review and editing. All authors contributed to the article and approved the submitted version.

## Funding

The study was funded by a grant to BS from the UKE Stiftung. This research was also funded by FEDER, Programa Interreg VA España-Portugal (POCTEP), Instituto Internacional de Investigação e Inovação em Envelhecimento–Capitaliza “0786_CAP4ie_4_P”. The funding sources had no role in the preparation of the data or manuscript.

## Conflict of interest

The authors declare that the research was conducted in the absence of any commercial or financial relationships that could be construed as a potential conflict of interest.

## Publisher’s note

All claims expressed in this article are solely those of the authors and do not necessarily represent those of their affiliated organizations, or those of the publisher, the editors and the reviewers. Any product that may be evaluated in this article, or claim that may be made by its manufacturer, is not guaranteed or endorsed by the publisher.
